# Patient Satisfaction, Side Effects, and Other Reactions Reported by Adult Men Prescribed Compounded Topical Finasteride via a National Telehealth Platform: Retrospective Analysis of Real-World Data

**DOI:** 10.2196/84676

**Published:** 2026-01-22

**Authors:** Jessica Yu, Sachie Mochida, Michele Emery, Patrick Carroll, Justin Ko, Arash Mostaghimi

**Affiliations:** 1 Hims & Hers Health, Inc, 2269 Chestnut Street, Suite 523, San Francisco, CA, 94123, United States, 1 6508048434; 2 Department of Dermatology, Stanford University School of Medicine, Stanford, CA, United States; 3 Department of Dermatology, Brigham and Women’s Hospital, Boston, MA, United States

**Keywords:** androgenetic alopecia, topical finasteride, topical minoxidil, patient satisfaction, side effects, telehealth

## Abstract

**Background:**

Topical minoxidil and oral finasteride are approved by the US Food and Drug Administration (FDA) for the treatment of male androgenetic alopecia (AGA). However, concerns about adverse events related to the use of oral finasteride have led to some apprehension about the treatment. Topical finasteride, though not FDA-approved, has demonstrated efficacy and safety in a limited number of clinical trials and may be a promising alternative, such that compounding pharmacies and telehealth companies in the United States now offer access to topical finasteride for patients with AGA.

**Objective:**

This real-world, retrospective study is, to our knowledge, the largest study to date aimed to evaluate patient satisfaction and tolerability associated with the novel combinations of topical finasteride and topical minoxidil for the treatment of male AGA.

**Methods:**

We conducted a retrospective analysis of patient data collected during routine clinical follow-up via Hims & Hers, a direct-to-consumer health and wellness platform, between April 1, 2021 and April 30, 2025 to assess the frequency of side effects and other possible medication reactions associated with the use of compounded topical finasteride and minoxidil. Data were gathered from two sources: (1) a follow-up check-in sent to patients approximately 130 days following the initiation of treatment; (2) unprompted communications sent via in-app or web-based messaging from patients to their care team. Data about patient satisfaction with treatment, the frequency of any side effect, frequency of specific side effects, need for a higher level of care, and treatment discontinuation due to a side effect were extracted from the data sources.

**Results:**

A total of 638,629 male patients with AGA received a prescription for a compounded topical finasteride and minoxidil product between April 1, 2021 and April 30, 2025. Of 151,352 (23.7%) patients who completed a follow-up check-in, 121,615 (80.4%) reported being satisfied with treatment and 4034 (2.7%) reported experiencing a side effect. Of all the 638,629 patients, 230 (0.04%) sent their care team a message (outside of check-ins) indicating a side effect or other possible medication reactions. No patient reported seeking a higher level of care or discontinued treatment due to such an occurrence.

**Conclusions:**

Patients prescribed novel formulations of compounded topical finasteride and minoxidil for the treatment of AGA via a national telehealth platform reported satisfaction with the treatment and tolerated it well. The limitations of the study include the use of retrospective data and the lack of a control group, both of which preclude causal inference. Future research should include randomized controlled trials to assess the efficacy, safety, and tolerability of topical finasteride.

## Introduction

Androgenetic alopecia (AGA), commonly referred to as “male pattern baldness,” is the most common form of hair loss in men. It affects approximately 50% of men worldwide [1] and an estimated 50 million in the United States alone [2]. Although AGA is considered a physically benign medical condition, it is associated with notable psychological consequences including low self-esteem, body dissatisfaction, social anxiety, and reduced quality of life [3].

Topical minoxidil and oral finasteride are two treatments currently approved by the US Food and Drug Administration (FDA) for the treatment of AGA. Topical minoxidil is available in both 2% and 5% formulations; the 5% formulation has been shown to be significantly superior in increasing hair regrowth, with an earlier response to treatment and good tolerance [4]. Oral finasteride has been shown, in clinical trials, to be well tolerated and effective in stabilizing hair loss and promoting hair growth [5]; however, reports of certain treatment-related adverse events such as sexual side effects and depression have led to some apprehension about the treatment, which may be negatively affecting the number of individuals who could benefit from it [6]. Notably, recent studies have questioned the purported causal relationship between oral finasteride and psychiatric symptoms [7,8].

Topical finasteride may be a promising alternative to oral finasteride. Though limited in number, studies that have examined the use of topical finasteride in the treatment of AGA have found it to be an effective and safe treatment option [9]. Two randomized controlled trials (RCTs) found topical finasteride to significantly decrease the rate of hair loss and significantly improve hair count compared to the placebo, with no differences in the incidence of adverse events or treatment discontinuation between the two groups [10,11]. Plasma concentrations of finasteride were 100-fold lower with the topical application of 0.25% finasteride spray versus 1 mg oral finasteride [11]. Furthermore, a systematic review of available RCTs, prospective studies, and retrospective medical record reviews found topical finasteride, either alone or in combination with other agents including topical minoxidil, to be non-inferior to oral finasteride and well-tolerated by patients—with the authors calling for larger cohort studies to examine the potential adverse event profile of the drug [9].

Unlike oral finasteride, topical finasteride is not currently FDA-approved for the treatment of AGA. It is, however, available as a compounded medication for those who do not want to take an oral medication or might be concerned about the reported side effects associated with oral finasteride. Several compounding pharmacies and telehealth companies in the United States now offer access to topical finasteride for patients with AGA. This real-world retrospective study is, to our knowledge, the largest study to date on patient satisfaction and tolerability associated with novel combinations of topical finasteride and topical minoxidil for the treatment of male AGA. We review anonymized patient data collected during the course of routine clinical care via a direct-to-consumer telemedicine platform to understand the patient-reported satisfaction and frequency of side effects and other possible medication reactions associated with compounded topical finasteride use (compounded topical finasteride is not FDA-approved or evaluated for safety, efficacy, or quality by the FDA).

## Methods

### Study Overview

Hims & Hers is a direct-to-consumer health and wellness platform that aims to increase access to treatment for adults aged 18 years and older with traditionally stigmatized conditions, including hair loss. Prospective patients seeking hair loss treatment come to the platform and complete a comprehensive clinical intake. Once the intake process is complete, a licensed medical provider thoroughly reviews the information gathered during the intake process, including medical history and treatment preferences, and has the opportunity to follow-up with the patient with any questions or remaining information deemed necessary to provide care. The provider then makes an independent clinical determination as to whether treatment is appropriate, and, if appropriate, shares a diagnosis and treatment plan. All licensed medical providers furnishing care through the platform are employed or contracted by You Health, a professional corporation owned and managed by licensed health care providers, which is the provider network associated with the platform. Patients sign up for a subscription to receive their medication dispensed by a licensed pharmacy at regular intervals. With this subscription, patients have ongoing, unlimited access to their care team via messaging and are sent follow-up check-ins to assess their treatment experience.

As of June 2025, three compounded topical finasteride and minoxidil products were available via the Hims & Hers platform to treat adult men with AGA: a spray consisting of 0.3% topical finasteride and 6% minoxidil, to be sprayed four times on the individual’s affected scalp area once per day; a spray consisting of 0.3% topical finasteride, 7% minoxidil, 2.2% ketoconazole, and 0.2% biotin, to be sprayed four times on the individual’s affected scalp area once per day; and a serum consisting of 0.3% topical finasteride and 6% minoxidil, 1 mL of which to be massaged into the individual’s affected scalp area once per day. All patients prescribed a compounded topical finasteride and minoxidil product were made aware that the product was not FDA-approved and were provided with instructions for use as well as education regarding what to expect with the treatment, common side effects, and other precautions. Patients also had access to educational treatment information via the Hims & Hers app and could contact their care team at any time with questions or concerns. In April 2025, the FDA issued an alert to health care providers, compounders, and consumers regarding potential risks associated with the use of compounded topical finasteride. This information was also shared with patients to ensure transparent communication regarding the products available through the platform.

To assess the frequency of side effects and other possible medication reactions associated with the use of compounded topical finasteride and minoxidil available via the Hims & Hers platform, we conducted a retrospective analysis of patient data collected during the course of routine clinical follow-up via the platform between April 1, 2021 and April 30, 2025. As this was an analysis of data gathered from individuals actively engaged in treatment, there was no control group.

### Data Collection

The analysis included two sets of data. The first set of data consisted of responses to a follow-up check-in assessment sent to patients approximately 130 days following treatment initiation. The check-in queried patients about their treatment satisfaction and experience with side effects. To assess treatment satisfaction, patients were asked to indicate “yes” or “no” to the following prompt: “I’m happy with the way my treatment is working.” To assess experience with side effects, patients were asked to respond “yes” or “no” to the following question: “Are you bothered by any side effects or other negative reactions from your treatment?” No other questions pertaining to side effects were included in the check-in.

The second set of data consisted of unprompted communications sent via in-app or web-based messaging from patients to their care team. Patients can send these unprompted messages at any time for review by the care team. These communications undergo continuous quality assurance by a clinical quality team that monitors patient messages in real-time for mention of side effects or other possible medication reactions and follows-up as appropriate. Their work includes validating the data to ensure that such events are appropriately recorded—for example, that the side effects and reactions reported are reported by patients in relation to one of the topical finasteride and minoxidil products highlighted in this analysis. Utilizing both sets of data ensured that all occurrences, both solicited and spontaneously reported by patients, were included in the analysis.

### Statistical Analysis

Descriptive statistics using Google Colab (Mountain View, CA) were used to quantify the percentage of patients who reported satisfaction with treatment in their follow-up check-in, the percentage of patients who reported having been bothered by side effects or other negative reactions in their follow-up check-in, the percentage of patients who indicated experiencing a side effect or other possible medication reaction in messages to their care team, the percentage of patients who sought a higher level of care due to such a reaction, and the percentage of patients who discontinued treatment due to such a reaction. For results regarding the percentages of patients who reported treatment satisfaction and side effects in their follow-up check-in, the number of patients who completed a check-in is used as the sample size. For results regarding the percentage of patients who reported a side effect to their care team, the total number of patients prescribed a compounded topical finasteride product is used a the sample size. This is due to the fact that all patients had the ability to message their care team; thus, all patients can be included in the denominator.

### Ethical Considerations

This study was approved by the WCG Institutional Review Board (Protocol 001, Review 20244102). All study procedures were conducted in accordance with the principles of the Declaration of Helsinki. The study protocol included a Waiver of Informed Consent, as all data analyzed were collected during the course of routine care and de-identified prior to analysis. Patients were not compensated for their participation in this study.

## Results

### Baseline Demographics

A total of 638,629 male patients with AGA received a prescription for a compounded topical finasteride and minoxidil product between April 1, 2021 and April 30, 2025. A total of 151,352 completed the follow-up check-in querying patients about their treatment satisfaction and experience with side effects.

The mean (SD) age of all patients who received a prescription for a compounded topical finasteride product (n=638,629) was 39.6 (11.9) years, while the mean (SD) age of those who completed the follow-up check-in (n=151,352) was 41.2 (11.8) years.

### Treatment Satisfaction and Side Effects as Reported During Follow-Up Check-In

Overall, 121,615 (80.4%, n=151,352, 95% CI [80.2%, 80.6%]) patients who completed the follow-up check-in reported being satisfied with their treatment. A total of 4034 (2.7%, n=151,352, 95% CI [2.6%, 2.8%]) reported experiencing side effects.

Of the 151,352 patients who completed the follow-up check-in, 138,645 had been prescribed the 0.3% topical finasteride and 6% minoxidil spray; 10,774 had been prescribed the 0.3% topical finasteride, 7% minoxidil, 2.2% ketoconazole, and 0.2% biotin spray; and 1933 had been prescribed the 0.3% topical finasteride and 6% minoxidil serum. Table 1 outlines treatment satisfaction and the frequency of side effects reported by patients receiving each treatment.

**Table 1.** Treatment satisfaction and frequency of side effects reported by patients during follow-up check-ins.

	All topical finasteride treatments (n=151,352)	Topical finasteride (0.3%) and minoxidil (6%) spray (n=138,645)	Topical finasteride (0.3%), minoxidil (7%), ketoconazole (2.2%), and biotin (0.2%) spray (n=10,774)	Topical finasteride (0.3%) and minoxidil (6%) serum (n=1933)
Treatment satisfaction, n (%)	121,615 (80.4)	111,165 (80.2)	8900 (82.6)	1550 (80.2)
Experienced side effects, n (%)	4034 (2.7)	3716 (2.7)	251 (2.3)	67 (3.5)

### Side Effects and Other Possible Medication Reactions Reported in Patients’ Communications to Their Care Team

Of the 638,629 patients prescribed a compounded topical finasteride and minoxidil product, 230 (0.04%, n=638,629, 95% CI [0.035%, 0.045%]) sent their care team messages concerning side effects or other possible medication reactions. The most commonly reported occurrences were scalp irritation (46/638,629, 0.007%, 95% CI [0.0064%, 0.0076%]), dizziness (33/638,629, 0.005%, 95% CI [0.0045%, 0.0055%]), increased heart rate (21/638,629, 0.003%, 95% CI [0.0026%, 0.0035%]), rash or some allergic reaction (19/638,629, 0.003%, 95% CI [0.0026%, 0.0035%]), and headache (18/638,629, 0.003%, 95% CI [0.0026%, 0.0035%]). Sexual side effects, specifically decreased libido and erectile dysfunction, were reported by 12/638,629 patients (0.002%, 95% CI [0.0017%, 0.0023%]). Depression was reported by 13/638,629 patients (0.002%, 95% CI [0.0017%, 0.0023%]). Anxiety was reported by 10/638,629 patients (0.002%, 95% CI [0.0017%, 0.0023%]). Cognitive concerns were reported by 10/638,629 patients (0.002%, 95% CI [0.0017%, 0.0023%]).

**Figure 1.**
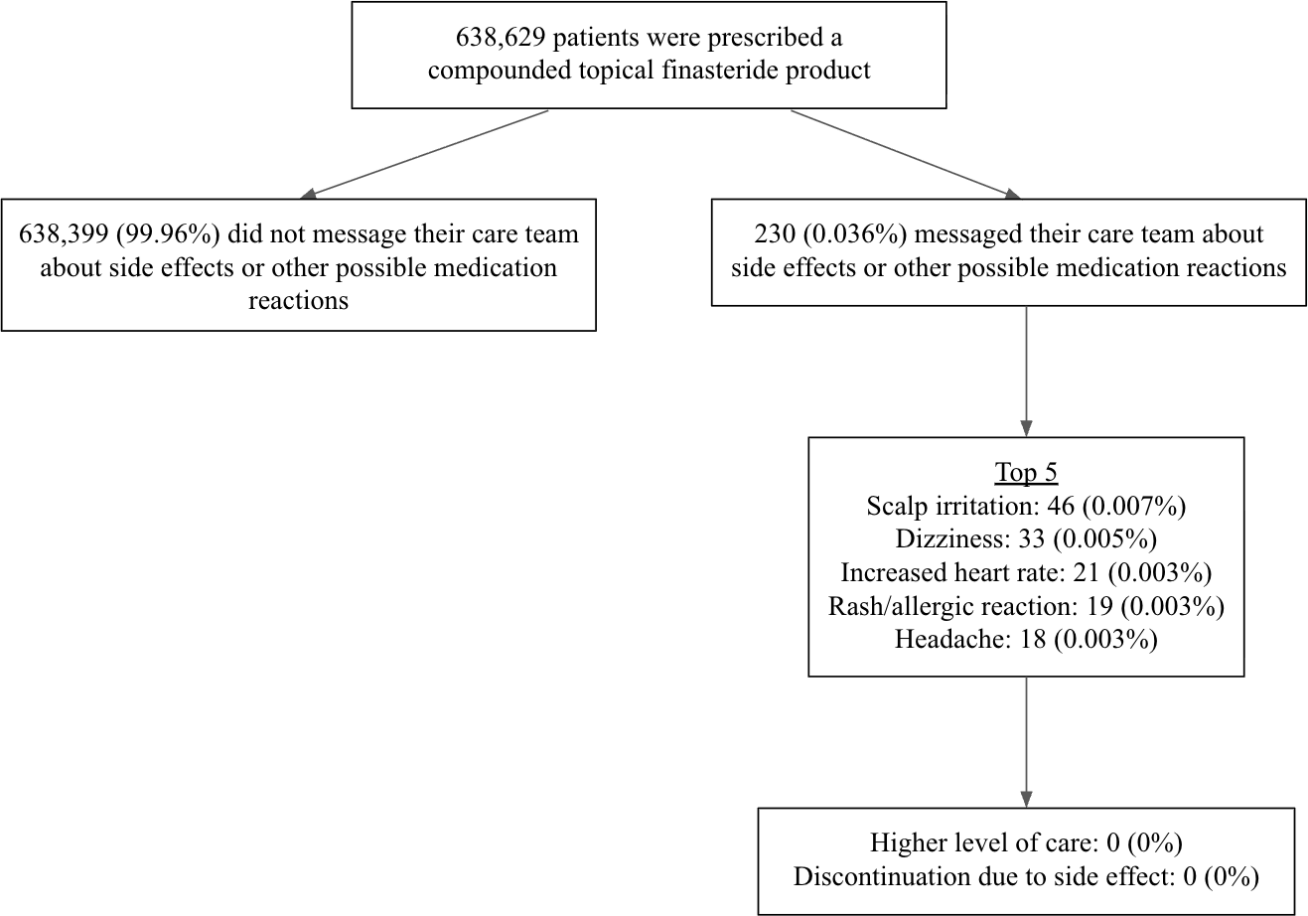
Schematic of side effects and other possible medication reactions reported by patients in messages to their care team.

No patients reported seeking a higher level of care (eg, emergency room or urgent care visit) related to a side effect or other possible medication reaction. No patients reported discontinuing treatment due to such an occurrence. During the study period, 1 spouse reported the death of a partner. Upon follow-up, no cause was identified and no causality was established. Figure 1 provides a summary of the side effects and other possible medication reactions reported by patients via messaging.

## Discussion

In this largest study of patient satisfaction and tolerability associated with the use of novel compounded formulations of topical finasteride and minoxidil, we found that 80% of those who completed a follow-up check-in reported satisfaction with treatment and less than 3% reported experiencing side effects. An additional 0.04% of patients sent their care team messages concerning side effects or other medication reactions. The most common reactions appeared to fall into one of two categories: (1) scalp irritation and rash, likely associated with the route of administration; (2) dizziness, increased heart rate, and headache, likely attributable to minoxidil acting as a vasodilator. Of note, sexual side effects, depression, anxiety, and cognitive concerns previously associated with oral finasteride were reported by just 0.002% of patients. There were no reports of “post-finasteride syndrome” [12].

Early clinical trials of 1 mg oral finasteride for the treatment of male AGA found that 3.8% of participants experienced adverse events possibly, probably, or definitely related to treatment, specifically decreased libido, erectile dysfunction, and ejaculation disorder, and 1.4% discontinued treatment due to such adverse events [13]. Trials of 2% topical minoxidil for the treatment of male AGA found that the most common adverse events were minor respiratory events such as colds and respiratory infections (3.37% of participants), followed by dermatological reactions such as itching (1.94%) [14]. Trials of 5% topical minoxidil for the treatment of male AGA found that headache was the most frequently reported adverse drug reaction (1.7%), followed by dermatological reactions such as pruritus (1.1%) and rash (1.1%) [14].

A comparison of our findings to the findings of these historic studies reinforces the favorable tolerability profile of topical medications. Altogether, these results demonstrate that the novel compounded formulations of topical finasteride and minoxidil available to male patients with AGA via the Hims & Hers platform are associated with high satisfaction among patients and few reported side effects.

To date, few clinical trials have examined the use of topical finasteride in the treatment of male AGA [10,11,15]. A Phase III RCT by the Topical Finasteride Study Group in Europe found that 41.4% of participants reported treatment-emergent adverse events and 9.9% experienced treatment-related adverse events [10]. Another Phase III RCT in China found that 68.4% of participants reported treatment-emergent adverse events and 8.3% experienced treatment-related adverse events [11]. In both studies, the frequency of adverse events among participants using topical finasteride was similar to those using placebo. A retrospective study of 238 patients who received topical finasteride via a German direct-to-consumer teledermatology platform and completed a 6-week follow-up questionnaire found that 11.8% of patients reported adverse events after initiating the use of topical finasteride [15].

However, the aforementioned studies are methodologically limited by their relatively small sample sizes. This study, which included over 600,000 patients who were prescribed compounded topical finasteride in a real-world context, offers a much more robust and meaningful assessment of patient-reported satisfaction and tolerability associated with treatment.

There are limitations of this analysis. First, this was a retrospective analysis of data collected during the course of routine care and not an RCT, and therefore, we cannot confirm any causal relationships between patients’ use of compounded topical finasteride and minoxidil and the reported outcomes. Second, we partly relied on data from an optional follow-up check-in questionnaire sent to patients approximately 130 days after treatment initiation. The rate of check-in completion was relatively low, with 23.7% of patients completing the check-in. This may indicate some selection bias, such that patients who were more engaged in or satisfied with their treatment may have been more likely to respond to the check-in and less likely to report side effects. Patients who reported side effects or other reactions to outside health care providers may not have been captured. Third, our reliance on retrospective data meant that we were unable to systematically examine other data of interest, such as the severity of and types of intervention sought for side effects and other medication reactions reported by patients.

However, our analysis also had several strengths. First, our sample size was impressive, with 638,629 patients prescribed a compounded topical finasteride product, all of whom had the ability to communicate with their care team at any time during the course of treatment, and 151,352 of whom completed the follow-up check-in that specifically queried patients about their experience with treatment and side effects. Second, our analysis utilized real-world data. The use of real-world data enables clinicians and researchers to better understand how patients experience treatment in their daily lives, thus increasing the generalizability of results. Third, in addition to relying on the optional follow-up check-in questionnaire to collect data on patient-reported side effects, we were also able to utilize unsolicited patient communications concerning side effects and other possible medication reactions. Having these additional data increased the likelihood that we were able to capture all occurrences reported by patients.

In conclusion, our analysis found that patients prescribed novel formulations of compounded topical finasteride and minoxidil for the treatment of AGA via a national telehealth platform tolerated the treatment well. The majority reported satisfaction with the treatment, and there were few reports of side effects. Future research should include RCTs to assess the efficacy, safety, and tolerability of topical finasteride. Together, this work may help provide more treatment options for those with AGA.

## ABBREVIATIONS

AGA: androgenetic alopecia
FDA: Food and Drug Administration
RCT: randomized controlled trial
